# An Odor Interaction Model of Binary Odorant Mixtures by a Partial Differential Equation Method

**DOI:** 10.3390/s140712256

**Published:** 2014-07-09

**Authors:** Luchun Yan, Jiemin Liu, Guihua Wang, Chuandong Wu

**Affiliations:** School of Chemistry and Biological Engineering, University of Science and Technology Beijing, Xueyuan Road 30, Haidian District, Beijing 100083, China; E-Mails: yanluchun@126.com (L.Y.); ghwang@ustb.edu.cn (G.W.); dong1035@126.com (C.W.)

**Keywords:** human sensing, monitoring, odor intensity, odor interaction, arenes, air pollution

## Abstract

A novel odor interaction model was proposed for binary mixtures of benzene and substituted benzenes by a partial differential equation (PDE) method. Based on the measurement method (tangent-intercept method) of partial molar volume, original parameters of corresponding formulas were reasonably displaced by perceptual measures. By these substitutions, it was possible to relate a mixture's odor intensity to the individual odorant's relative odor activity value (OAV). Several binary mixtures of benzene and substituted benzenes were respectively tested to establish the PDE models. The obtained results showed that the PDE model provided an easily interpretable method relating individual components to their joint odor intensity. Besides, both predictive performance and feasibility of the PDE model were proved well through a series of odor intensity matching tests. If combining the PDE model with portable gas detectors or on-line monitoring systems, olfactory evaluation of odor intensity will be achieved by instruments instead of odor assessors. Many disadvantages (e.g., expense on a fixed number of odor assessors) also will be successfully avoided. Thus, the PDE model is predicted to be helpful to the monitoring and management of odor pollutions.

## 1. Introduction

Odor measurement is essential for odor control and management, and odor sensory methods are normally employed except for some instrumental methods. Sensation of odor is usually evaluated from four aspects including odor intensity, odor concentration, odor quality and hedonic tone. Odor intensity (OI) is the perceived strength of odor sensation. It can indirectly reflect the concentration level of either an individual compound or a complex mixture [1]. In the field of air pollution, most of related control standards mainly focus on the concentration limits of several targeted contaminants [2,3]. But, the air still always has distinct smell even though all the targeted contaminants are qualified to the restrictions. It is usually attributed to the odor interaction among odorants. Several typical kinds of odor interaction (synergism, antagonism, averaging effect, *etc.*) have already been researched and reported in forms of various models [4,5]. These models mainly focus on methods relating individual odorants to their joint odor property (*i.e.*, odor quality, odor concentration and odor intensity). But for ordinary people without specialized knowledge, describing and evaluating odor pollution level with OI is much easier to understand and more intuitive than both chemical concentration and odor concentration [6]. After a prolonged exposure to a particular odor, assessors will become unable to distinguish due to olfactory fatigue (olfactory adaptation) [7,8]. Therefore, it is almost infeasible to measure OI both from source emissions and in the ambient air. Generally, odor intensity is rated by a panel of professional odor assessors in a specific testing room. In order to expand the application of odor intensity, the participation of odor assessors is supposed to be displaced in the measurement of odor intensity. Because of that, an odor interaction model relating individual components to their joint odor intensity is needed. At present, several models have been proposed and they mainly focus on the relationship between the perceived odor intensity of a mixture and the individual odor intensities of its unmixed components [9]. In order to get rid of odor assessors, the individual odor intensities of each unmixed component in the above mentioned odor interaction models have to be displaced. If the odor intensity of a mixture is related to chemical concentrations of its components, odor intensity of a mixture can be directly calculated (*i.e.*, prediction of odor intensity) after quantitative analysis. Thus, OI can be better applied in the monitoring and management of odor pollution by combining with portable gas detectors and on-line monitoring systems.

For odor mixtures, odor interactions are considered to be complex and have not yet been fully understood. When investigating the solution volume of liquid mixtures, similar interactions also exist, but by using a set of partial differential equations, these interactions are successfully explained with partial molar volume [10]. For example, the influence of constituents' mixing ratio to the volume of an ethanol-water solution can be analyzed from the change of corresponding partial molar volumes of both ethanol and water. Comparing with both the linear concentration-volume relationship of individual liquid compounds and interaction characters of liquid mixtures, similar phenomena are also generally observed when exploring the concentration-OI relationship of both individual odorant and odor mixtures [4,11]. Based on these similarities between odor mixtures and liquid mixtures, odor interactions can be investigated by referencing them to the partial molar volume measurement method.

In this paper, benzene and substituted benzenes are chosen as the targeted compounds. It is not only because of their general existence in many areas, but also because of their related structures as they are not really strong odorants. A series of laboratory tests had been conducted to investigate the odor interaction of binary odor mixtures by a partial differential equation (PDE) method. The experimental data were then used to establish PDE models for specific binary mixtures and an extended PDE model. Besides, their OI predictive performances were verified through a series of odor intensity matching tests. Based on these results, the possible application of PDE model in odor control and management was discussed.

## 2. Experimental Section

### 2.1. Stimuli and Assessors

The following odorants were used: benzene (B, 99.5%), toluene (T, 99.5%), ethylbenzene (E, 98.5%), *n*-propylbenzene (NP, 98.5%), *o*-xylene (OX, 98%), *m*-xylene (MX, 98%) and styrene (S, 98%). All the stimuli were purchased from J&K Scientific (Beijing, China).

Fifteen assessors from the University of Science and Technology Beijing were recruited. Their ages ranged from 20 to 35 years. All of them had passed the olfactory selection test and participated in several experiments using the same procedures and apparatus [12]. All the assessors were divided into panel A (five males and five females) and panel B (three males and two females). Panel A was mainly employed in the tests for establishing PDE models, and the panel B was only employed in the odor intensity matching tests.

### 2.2. Measurement of Odor Threshold and Odor Intensity

Odor thresholds (the lowest concentration of an odorant that is perceivable by human sense of smell) were measured by the Force-Choice method using a dynamic olfactometer (AC'SCENT, St. Croix Sensory, Inc., Stillwater, MN, USA). An odorant with known concentration was delivered by the olfactometer in an ascending dilution series. Assessors sniffed from the sniffing mask with one presentation of dilute odor and two blank presentations in turn. The ascending dilution series were chosen mainly for the purpose of recognizing targeted odor quality at the beginning of test and then better distinguishing the targeted odor from blank samples at relative lower concentration levels. The possible olfactory fatigue after sniffing strong odors could be successfully diminished with sufficient interval time (about 30 s) between two continuous dilution levels. Until an incorrect decision occurred, the test finished and corresponding dilution multiple was recorded. After all the assessors completed the tests, odor threshold was calculated from the initial concentration of the odorant and average of all the recorded dilution multiples. In this study, the odor threshold of each odorant was measured by assessors in panel A. As shown in Table 1, the measured odor thresholds and other two sets of reported odor thresholds are listed. Through comparison, the measured odor thresholds were similar with the reported values. Because the composition of a sensory panel (e.g., quantity, age and gender) usually influences the measurement of odor threshold, differences between the two sets of reported odor thresholds are generally accepted. Thus, the measured odor thresholds in this study were accurate and it would be employed in the following calculations.

Before the preparation of odor samples, each odorant was respectively injected into a primary odor bag (3 L volume and full of odor-free air). When all the odorants had completely evaporated, odor sample was prepared through transferring a certain amount of gas from the primary odor bags to a new odor bag by syringe. Before test, three minutes' standing at room temperature was necessary for each odor sample. Water solutions of 1-butanol were prepared at 25 ± 1 °C according to the odor intensity referencing scale (OIRS, from level 1 (aqueous solution of 12 ppm) to level 8 (1550 ppm) with a geometric progression of two) [15]. During test, assessor gently pushed the odor bag and odor ran out from a glass pipe (inner diameter of 1 cm) on the bag with a quite small flow rate. Nose of assessor was normally about 5 cm away from the glass pipe. Thus, assessors inhaled the odor from bag almost the same as in the ambient air. Then, assessor was asked to find a scale point on the OIRS causing the olfactory stimulation as strong as the odor sample. The difference of odor qualities between the odor sample and 1-butanol should be ignored. Besides, assessor was permitted to check and recheck both the odor sample and 1-butanol solutions until they confidently found the best match on the OIRS. If the best match on the scale was a position between two scale points, then a half number (e.g., 6.5) was used. For each odor sample, its odor intensity was calculated as the average of all the scores rated by assessors in a corresponding panel.

### 2.3. Establishment Method of the PDE Model

As previous mentioned, odor interaction is supposed to be investigated by referencing to the measurement of partial molar volume. Usually, the partial molar volume is measured by a set of formulas (*i.e.*, tangent-intercept method). For example, the tangent-intercept method for an ethanol-water solution is described below [16]

(1) $$V_{\text{m}}=V/(n_{\text{Eth}.}+n_{\text{Wat}.})$$

(2) $$x_{\text{Eth}.}=n_{\text{Eth}.}/(n_{\text{Eth}.}+n_{\text{Wat}.})$$

where *V* is the volume of ethanol/water solution, *V*_m_ is the molar volume, *n*_Eth._ (or *n*_Wat._) is the number of moles, and *x*_Eth._ (or *x*_Wat._) is the molar proportion of ethanol (or water). For graphing purpose, let *V*_m_ be the vertical axis and *x*_Eth._ be the horizontal axis. Then, an ethanol-water solution will be presented as a point on the graph. If all the ethanol-water solutions with various mixing ratios are tested, their corresponding points will be connected to be a continuous curve (e.g., *y* = *f*(*x*)). Plotting a tangent line (e.g., *y*_2_ = *f*_2_(*x*)) at a point on the curve, its intercepts on the two vertical axis (*i.e.*, *x* = 0 and *x* = 1) are the corresponding partial molar volumes. Detailed calculation equations are described below [16]:

(3) $$y_{2}=f_{2}\left(x\right)=\left(x-x\prime \right)\cdot f\prime \left(x\prime \right)+y\prime$$

(4) $$V_{\text{Eth}.,\text{m}}=f_{2}\left(1\right)=\left(1-x\prime \right)\cdot f\prime \left(x\prime \right)+y\prime$$

(5) $$V_{\text{Wat}.,\text{m}}=f_{2}\left(0\right)=-x\prime \cdot f\prime \left(x\prime \right)+y\prime$$

where *y*_2_ = *f*_2_(*x*) is the tangent line at point (*x′*, *y′*), *V*_Eth.,m_ (or *V*_Wat.,m_) is the partial molar volume of ethanol (or water) when *x*_Eth._ = *x′*.

The establishment method of PDE model is firstly to displace these parameters (e.g., *V*, *n*_Eth._ and *n*_Wat._) of the above partial molar volume formulas with suitable perceptual measures. After a series of sensory tests for binary mixtures of benzene and substituted benzenes, experimental data will be calculated and depicted like the above tangent-intercept method. Through nonlinear regression analysis for these depicted points, it is planned to identify whether it is feasible to investigate odor interaction by referencing to the measurement method of partial molar volume. If it is feasible, the PDE model will be successfully established as a set of partial differential equations. In this study, all the nonlinear regressions were conducted using PASW statistics 18 software (IBM Co., New York, NY, USA).

### 2.4. Experimental Procedure

This study was consisted of three parts. In the first part, assessors in panel A explored the linear relation between OI and concentration of individual odorant. Then, original parameters of partial molar volume formulas were suitably displaced by perceptual measures. In the second part, four binary mixtures of benzene and substituted benzenes were individually tested. Each binary mixture was prepared as 22 different odor samples with various mixing ratios and different concentration levels. The OI of each odor sample was rated by the panel A. According to the results in the first part, a PDE model was respectively established for each odor mixture. After that, several new odor samples were prepar ed and continuously rated by assessors in panel B. At the same time, their odor intensities were also calculated on the basis of corresponding PDE models and measured chemical concentrations. Through odor intensity matching tests, the predictive performance of each PDE model was proved. In the third part, data of the above four PDE models were gathered and an extended PDE model was established. Based on that, odor intensity matching tests were performed for a new set of binary odor samples. Finally, the predictive performance and feasibility of the extended PDE model were discussed in detail.

In order to avoid too strong olfactory stimulation, all the odor samples of both individual odorants and binary mixtures were prepared below 7.0 of the OIRS (odor intensity referencing scale). For odor samples of the individual odorants in the first part test, the chemical concentration range was 5.8 to 194.2 g/m^3^ of benzene, 5.8 to 96.2 g/m^3^ of toluene, 0.9 to 48.2 g/m^3^ of ethylbenzene and 2.9 to 48.7 g/m^3^ of *o*-xylene. All the odor samples of binary mixtures were prepared by odorants included in the above chemical concentration ranges. The specific concentration of each odor sample was measured by a gas chromatography (GC-2014, Shimadzu, Kyoto, Japan) with a flame ionization detector (GC-FID) and a Rtx-5 capillary column (30 m × 0.25 mm ID, 0.5 μm film thickness). The carrier gas was nitrogen (≥99.999%) at 1.0 mL/min and the injection port was 200 °C. The column oven temperature was set to 50 °C for 3 min and up to 200 °C at 10 °C·min^−1^ and held for 5 min.

## 3. Results and Discussion

### 3.1. Key Parameters of the PDE Model

For an ethanol-water solution, the partial molar volume is employed to analyze the relationship between its solution volume and molar numbers of its constituents. Thus, both *V* and *n* are key and fundamental parameters in the measurement of partial molar volume (Equations (1) and (2)). For a binary odor mixture, the PDE model is also supposed to link a binary odor mixture's OI with its constituents' concentrations. If investigating the odor interaction by simulating the partial molar volume formulas, *V* in Equation (1) must be corresponding to the OI of an odor mixture. However, the corresponding variable of *n* is uncertain. Because the volume of an individual liquid substance has a strict linear relation with its molar number, and that is considered to be an important precondition of partial molar volume. In order to successfully establish the PDE model, the corresponding variable of *n* shall also have a linear relation with the OI of individual odorants. Because the chemical concentration and OI of individual odorants had been proved following the Weber-Fechner law (a nonlinear relation), other possible physical quantities were tested [17]. It had been reported that OI of an individual odorant had a linear relation with its logarithm of dilution-to-threshold (D/T) ratio (*i.e.*, log (D/T)) [11]. The D/T ratio actually means the dilution multiples of an odor sample when it is diluted to odorless. In theoretical, the D/T value of an odor sample is equal to its odor concentration and odor activity value (OAV). However, both the D/T ratio and odor concentration are normally measured by a sensory panel. But OAV can be calculated on the basis of corresponding odor threshold and chemical concentration. Thus, whether OAV was a suitable corresponding variable of *n* in the partial molar volume formulas was tested.

**Figure 1.**
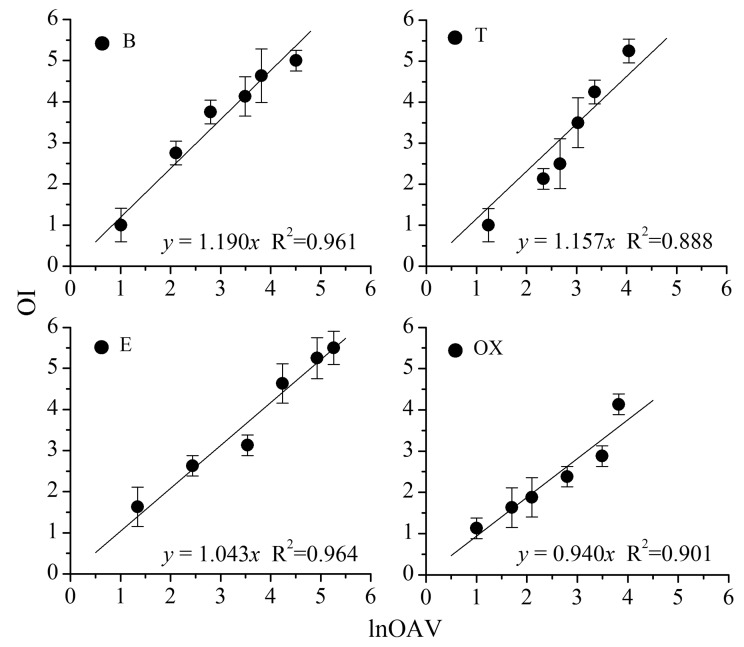
Linear relation between OI and lnOAV of individual benzene (B), toluene (T), ethylbenzene (E) and *o*-xylene (OX).

As shown in Figure 1, a significant linear relation is found between OI and lnOAV (natural logarithm of odor activity value). Each point on the figure indicates an odor sample, and the corresponding OI is the average of ten measured scores (ten assessors in panel A). The linear regression equations and R^2^ values are also provided. The OAV is a ratio between an odorant's chemical concentration and its odor threshold (*i.e.*, OAV = C/C_Thr._). Because odor threshold is normally used as a constant, OAV can be directly calculated from the measured chemical concentration. Thus, OAV is indirectly on behalf of the chemical concentration and lnOAV just is its mathematical transformation. Based on the similarity between *n* and lnOAV, *n* in the above Equations (1) and (2) was considered to be displaced by lnOAV.

Based on the above results, specific calculation formulas of PDE model were established with referencing to the tangent-intercept method in Section 2.3:

(6) $${\text{OI}}_{\text{m}}=\text{OI}/(\ln{\text{OAV}}_{a}+\ln{\text{OAV}}_{b})$$

(7) $$x_{a}=\ln{\text{OAV}}_{a}/(\ln{\text{OAV}}_{a}+\ln{\text{OAV}}_{b})$$

(8) $${\text{OI}}_{a,\text{m}}=f_{2}\left(1\right)=\left(1-x\prime \right)\cdot f\prime \left(x\prime \right)+y\prime$$

(9) $${\text{OI}}_{b,\text{m}}=f_{2}\left(0\right)=-x\prime \cdot f\prime \left(x\prime \right)+y\prime$$

where *a* and *b* represent two different odorants; OI_m_ is the averaged OI, which is corresponding to *V*_m_; *x_a_* is the mixing proportion of compound *a*, which is corresponding to *x*_Eth._ (or *x*_Wat._); OI*_a_*_,m_ (OI*_b_*_,m_) is partial differential OI, which is corresponding to partial molar volume (*V*_Eth.,m_ or *V*_Wat.,m_).

### 3.2. PDE Models for Binary Mixtures of Benzene and Substituted Benzenes

**Figure 2.**
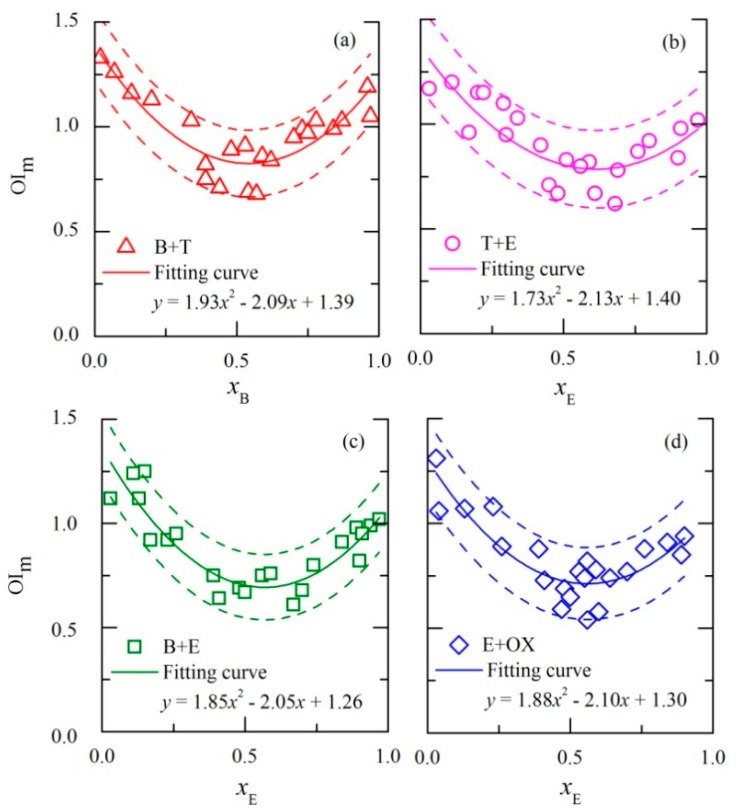
PDE models for mixture of: (**a**) benzene and toluene (B+T); (**b**) toluene and ethyl benzene (T+E); (**c**) benzene and ethyl benzene (B+E); (**d**) ethyl benzene and *o*-xylene (E+OX). Fitting curve (solid line) of 22 different odor samples and corresponding prediction intervals (dashed line, confidence interval was 0.90) are respectively depicted.

The PDE model of each binary odor mixture was individually plotted in Figure 2. Each point on the figure represents an odor sample and every binary mixture contains 22 different odor samples. Each odor sample was rated by the panel A (ten assessors) and average of the ten scores was calculated as its measured OI. The lnOAV of each odor sample was also calculated on the basis of its concentration and measured odor threshold (Table 1). Before establishing the PDE model, the measured OI and lnOAV were calculated according to Equations (6) and (7). Then, a series of nonlinear regressions (*i.e.*, quadratic, logarithmic, cubic, exponential, power and logistic models) were respectively made for each binary mixture. Through comparison, the quadratic polynomial regression method (a kind of nonlinear regression) showed the best matching effect and it was employed to plot the fitting curves of each mixture. As depicted in Figure 2, the fitting curve of each odor mixture looks like an asymmetric “U” shape. Finally, the PDE model of each binary odor sample was completely established.

As shown in Figure 2, the discipline of odor interaction can be distinctly observed from the shape of fitting curve. It is apparent to conclude that an odorant's influence to the OI of a binary odor mixture is proportional to its mixing ratio. For example of mixture B+T (Figure 2a), the tangent line on the fitting curve was nearly horizontal when *x*_B_ ≈ 0.5. Then, OI_B,m_ (intercept on vertical axis *x* = 1) and OI_T,m_ (intercept on vertical axis *x* = 0) are almost the same. It means that both odorants equally influenced the OI of mixture (Equation (11)). If *x*_B_ > 0.5, OI_B,m_ will be bigger than OI_T,m_. Then, the same dose of benzene causes more influence to the OI of mixture than toluene. Comparing with traditional odor interaction models (e.g., U model, Vector model, Strongest component model, Additivity model) about OI, the most important difference is PDE model expressing odor interaction in forms of a line graph [5,18]. It is not only a more interpretable way, but also shows more details about the odor interaction trends. Actually, the PDE model is more like the perfumery ternary diagrams (PTD) and perfumery quaternary diagrams (PQD) [19,20]. The PTD and PQD are developed basing on an analogy between perfume pyramid structure and engineering ternary/quaternary diagrams. Both the reported perfumery diagrams and PDE model in this study are empirical models, and used to predict the odor characters of odor samples. But PDE model is more focus on interpreting the odor interaction in a quantitative method. Every parameter in the PDE model has definite physical meaning. As shown in Equation (11), OI*_i_*_,m_ is easily comprehended as the OI of each unit (lnOAV) corresponding odorant. But it can be apparently observed that one of the OI*_i_*_,m_ values will be negative when the mixing ratio is close to 1.0. As it known, OI of any odorant is impossible to be negative. Thus, it is better to just comprehend OI*_i_*_,m_ as its definition (change of a mixture's OI when a lnOAV unit of corresponding odorant is added into the mixture).

For each binary odor mixture, the OI_m_ values are evenly distributed within 90 percent confidence interval of the regression curve (Figure 2). That's because odor intensity is a subjective feeling of the human olfactory system. Many influencing factors have been found and people's olfactory differences are widely accepted [21]. Thus, the OI of a same odor sample measured by various assessors is usually different. For example, a difference of within 0.5 between two measured OIRS scores is usually acceptable when a same odor sample is continuously rated twice. For a binary mixture at a low concentration level (the summation of two lnOAV is about 2.0 based on the experimental data), it (0.5 of the OIRS) causes a difference of 0.25 between two corresponding OI_m_ values (Equation (6)). For mixtures at high concentration levels (summation of lnOAV is about 5.0 to 7.0), the corresponding difference of OI_m_ values will be much smaller. Thus, the fluctuation of OI_m_ values (±0.2 around the regression curve) in Figure 2 was accepted.

Except for analyzing the odor interaction, the PDE model also can be used for OI prediction. As shown in Figure 2, the OI_m_ displays a tight relation with components' mixing ratio. Each point on the fitting curve corresponds to a binary odor sample with corresponding proportion on the horizontal axis. If the odor concentrations of two components have been measured, OI of a binary odor sample can be predicted through calculation on the basis of corresponding PDE model. For example, an odor sample of mixture B+T has the mixing ratio of 0.6 (lnOAV*_B_* = 3.36 and lnOAV*_T_* = 2.24). According to the regression equation (Figure 2a, *y* = 1.93*x*^2^ − 2.09*x* + 1.39), the location of this sample is (0.6, 0.83). Plotting a tangent line at the point on the regression curve, the corresponding equation of the tangent line is *y* = 0.23*x* + 0.72 (Equation (3)). According to Equations (8) and (9), its intercepts on the two vertical axis (*x* = 0 and *x* = 1) are the corresponding OI*_B_*_,m_ = 0.95 and OI*_T_*_,m_ = 0.72. Based on the summation formula of partial molar volume:

(10) $$V=V_{1,\text{m}}\cdot n_{1}+V_{2,\text{m}}\cdot n_{2}$$

and the odor intensity of a binary mixture can be calculated following the formula:

(11) $$\text{OI}={\text{OI}}_{a,\text{m}}\cdot \ln\text{OAV}a+{\text{OI}}_{b,\text{m}}\cdot \ln{\text{OAV}}_{b}$$

Thus, the OI of the above odor sample can be calculated as: OI = 3.36 × 0.95 + 2.24 × 0.72 = 4.8. Based on the above mentioned results, the OI of any binary mixture can be predicted on the basis of corresponding PDE model and instrumental measured chemical composition (*i.e.*, lnOAV of each component).

In order to demonstrate the predictive performance of PDE model, a new set of odor samples were prepared and rated by panel B (five assessors). The average of five measured scores was calculated for each odor sample as its measured OI. At the same time, OI of each sample (predicted OI) was also calculated by employing the corresponding PDE model. As shown in Table 2, the PDE model shows good predictive accuracy for a wide range of component proportions and concentration levels.

### 3.3. Extended PDE Model for Binary Mixture of the Benzene and Substituted Benzenes

**Figure 3.**
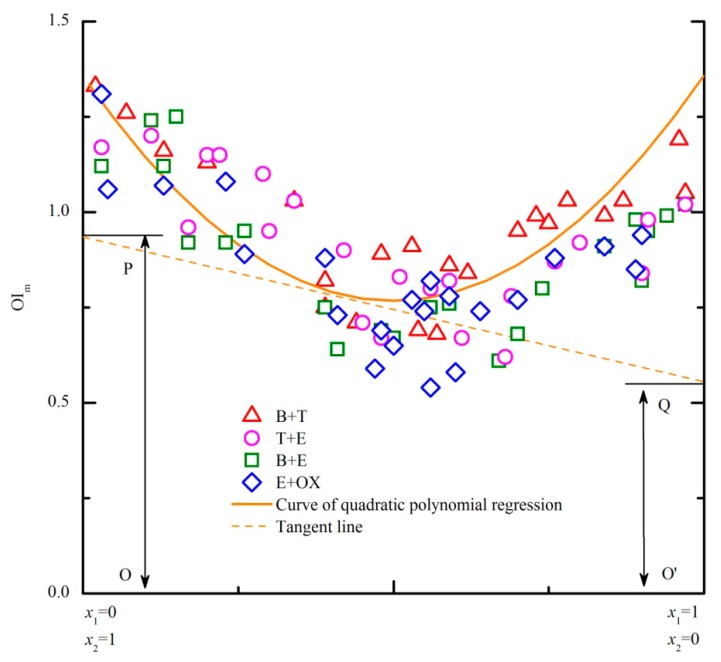
An extended PDE model for any binary odor mixture of benzene and substituted benzenes. Equation of the fitting curve (solid line) was *y* = 2.36*x*^2^ −=2.36*x* + 1.36. The dashed line was a tangent line to the curve at the given point (0.4, 0.8). Value of the intercept O′Q (or OP) equaled to OI_1,m_ (or OI_2,m_).

In the above experiment, each PDE model was related to a specific odor mixture. Based on the similarities among these four PDE models (Figure 2), a more commonly used model for any binary odor mixture of the benzene and substituted benzenes was expected. As shown in Figure 3, all the data of the four binary mixtures are gathered and an extended PDE model is established. Because all the regression curves of the original PDE models are asymmetric “U” shapes, the corresponding regression equations are closely related with the design of horizontal axis (Figure 2). If positions of the two components on the horizontal axis exchange, the corresponding regression equation is also different. Thus, the regression curve of the extended PDE model was adjusted to be symmetry (axis of symmetry is *x* = 0.5) for solving the problem (Figure 3). Even though relativity of the symmetric regression curve may be influenced, the applicable scope and usability of the extended PDE model will be apparently enhanced.

Odor samples of four binary odor mixtures (Table 3, part A) were prepared to test the predictive performance of the extended PDE model. Both new odorants (NP and MX) and previously used odorants were employed in the four mixtures. Each odor sample was rated by panel B (five assessors) and then average of five scores was calculated as the measured OI. The extended PDE model, U model, SC (strongest component) model and ADD (additivity) model were employed for the prediction of OI. But except for the extended PDE model, the other three models (U, SC and ADD model) mainly concerned on the relation between odor intensities of a mixture and its unmixed components. Thus, each odor sample's unmixed components were also rated by panel B in advance (Table 3, measured OI*_a_* and OI*_b_*). In order to compare the predictive performance of these models, average of all the samples' predictive coefficients (ratio of predicted OI and measured OI) was respectively calculated for each model. The predicted OI and measured OI were considered to be the same when the predictive coefficient was 1.0. As shown in Table 3, both the extended PDE model and U model have the closest predictive coefficients to 1.0. Besides, specific comparisons among several models had been reported and U model showed the best predictive accuracy [9]. Thus, predictive accuracy of the extended PDE model is proved well.

As a key parameter of U model, cos α was calculated on the basis of corresponding measured OI*_a_*, OI*_b_* and OI*_a_*_+_*_b_* (Table 3). Even belonging to a same odor mixture, the value of cos α was still different for different odor samples. Therefore, the predictive accuracy of the U model was seriously influenced by the measurement of cosα. However, the same extended PDE model was always usable for any binary odor mixture of the benzene and substituted benzenes. Thus, the extended PDE model is considered more feasible than U model.

In order to explore the possible applicable scope of the extended PDE model, an extra set of odor samples of two binary odor mixtures (T+S and E+S) were tested (Table 3, part B). The procedure of odor intensity matching test was the same with the above tests. As a result, the extended PDE model still maintained well predictive performance. Thus, it could be concluded that the extended PDE model was not only limited to the binary mixture of the benzene and substituted benzenes. Because styrene has the similar odor quality with the benzene and substituted benzenes, whether the applicable scope of extended PDE model is influenced by the molecular structure or odor quality of odorant is supposed to be explored in the further experiments.

## 4. Conclusions

In this paper, a novel odor interaction model was proposed for binary mixtures of benzene and substituted benzenes. The binary odor mixture was explored in forms of partial differential equations (PDE) by simulating the measurement method of partial molar volume. After establishing a specific PDE model for each individual binary mixture, an asymmetric U-shape of the OI_m_ *vs.* mixing ratio was distinctly observed. Form the U-shape, it was concluded that an odorant's influence to the OI of a binary odor mixture is proportional to its mixing ratio. Thus, the PDE model was considered to be a more interpretable way relating the concentration of each component to their joint odor intensity. In order to improve the applicable feasibility of PDE model, an extended PDE for any binary mixture of benzene and substituted benzene were also established. Besides, the PDE models were also employed for the prediction of odor intensity. After a series of odor intensity matching tests, the predictive accuracy and applicable feasibility of both the PDE models and extended PDE model were proved to be good. In a certain environment, chemical concentrations of targeted pollutants can be easily measured by employing appropriate portable detectors (or online monitoring systems). Then, the OI of an odor mixture can be directly predicted through calculation on the basis of corresponding established PDE model. For mixtures containing more than two components, a classification method is necessary to simplify its composition in advance. Thus, the classification method and PDE model for complex mixtures will be researched in our further experiments. If combining the PDE model with portable detectors or online monitoring systems, both chemical concentrations of targeted compounds and odor intensity of the ambient air can be immediately measured on site. Thus, the advantages of odor intensity evaluation will be fully utilized in more related fields.

## Figures and Tables

**Table 1.** List of stimuli investigated for odor interaction model.

**Order**	**Odorant (abbreviation)**	**CAS#**	**Chemical Structure**	**Reported Odor Threshold/mg/m3 (ppm)**	**Measured Odor Threshold ^III^/mg/m3 (ppm)**
1	Benzene (B)	71-43-2	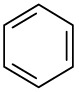	8.79(2.70) ^I^; 28.15(8.65) ^II^	2.13(0.66)
2	Toluene (T)	108-88-3	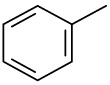	1.27(0.33) ^I^; 0.61(0.16) ^II^	1.67(0.43)
3	Ethylbenzene (E)	100-41-4	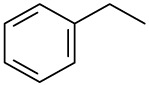	0.75(0.17) ^I^; 10.17(2.3) ^II^	0.25(0.056)
4	*o*-Xylene (OX)	95-47-6	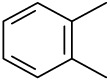	1.68(0.38) ^I^; 3.76(0.85) ^II^	1.07(0.24)
5	*n*-Propylbenzene (NP)	103-65-7	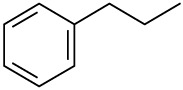	0.019(0.0038) ^I^	0.97(0.19)
6	*m*-Xylene (MX)	108-38-3	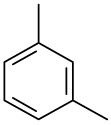	0.18(0.041) ^I^; 1.43(0.32) ^II^	1.35(0.31)
7	Styrene (S)	100-42-5	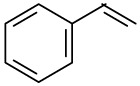	0.15(0.035) ^I^; 14.93(3.44) ^II^	0.39(0.090)

I Odor detection thresholds in reference [13];

II Odor detection thresholds in reference [14];

III Odor detection thresholds measured by panel A in this study.

**Table 2.** Key parameters of PDE model, and comparison between the measured OI and corresponding predicted OI.

**Mixture**	**Proportion**		**Regression Equation**	**OI***_a_***_,m_**	**OI***_b_***_,m_**	**Predict. OI**	**Measur. OI**

	**lnOAV***_a_*	**lnOAV***_b_*					
a = T b = E	3.36	3.54	*y* = 1.73*x*^2^ − 2.13*x* + 1.40	0.95	0.60	5.3	5.5
	4.05	1.75		1.25	0.16	5.3	5.5
	2.34	3.54		0.77	0.74	4.4	4.1
	2.67	2.44		1.01	0.54	4.0	3.6
	1.65	5.26		0.40	0.91	5.4	5.8

a = E b = OX	5.26	1.70	*y* = 1.88*x*^2^ − 2.10*x* + 1.30	0.96	0.23	5.5	6.0
	4.93	2.80		0.83	0.54	5.6	5.6
	3.54	3.49		0.61	0.82	5.1	4.7
	2.44	3.49		0.43	0.98	4.5	4.1
	1.34	3.82		0.05	1.17	4.5	4.6

**Table 3.** Comparisons of odor intensity predictive performance among the extended PDE model, U model, SC model and ADD model.

**Mixture**	**Measured OI**			**OI***_a_***_,m_**	**OI***_b_***_,m_**	**Predicted OI (a + b)**			
	
	**a**	**b**	**a + b**			**PDE**	**U**	**SC**	**ADD**
**A. Binary odorant mixture of the benzene and substituted benzenes**									

a = T b = E	4.3	3.1	5.5	0.70	0.76	5.0	4.7	4.3	7.4
	3.5	4.6	4.2	0.52	0.91	5.4	5.2	4.6	8.1
	2.1	3.1	4.1	0.46	0.95	4.4	3.4	3.1	5.2
	2.5	2.6	3.6	0.78	0.68	3.7	3.3	2.6	5.1
	2.1	2.6	3.0	0.71	0.75	3.5	3.0	2.6	4.7

a = E b = OX	5.3	2.9	6.4	0.91	0.51	6.3	5.9	5.3	8.2
	5.3	2.4	5.6	1.01	0.36	6.0	5.6	5.3	7.7
	3.1	2.9	4.7	0.74	0.72	5.1	4.3	3.1	6.0
	2.6	2.9	4.1	0.50	0.92	4.4	4.0	2.9	5.5
	3.1	2.4	3.9	0.86	0.58	4.7	4.0	3.1	5.5

a = E b = NP	2.6	6.0	6.2	0.49	0.93	4.5	5.4	6.0	8.6
	5.2	3.0	5.0	1.24	−0.26	5.8	5.0	5.2	8.2
	4.7	3.7	4.9	1.07	0.25	5.4	5.0	4.7	8.4
	3.1	3.7	4.1	0.97	0.42	4.4	4.0	3.7	6.8
	2.6	3.0	2.8	1.09	0.20	2.9	3.3	3.0	5.6

a = OX b = MX	6.3	2.0	6.2	1.12	0.14	5.3	6.4	6.3	8.3
	2.9	4.3	5.2	0.77	0.69	4.9	5.3	4.3	7.2
	4.0	3.4	5.1	0.94	0.47	4.8	5.4	4.0	7.4
	2.4	2.0	3.8	0.94	0.47	3.5	3.2	2.4	4.4
	1.5	3.4	3.7	0.61	0.84	3.4	3.7	3.4	4.9

Average of Predict./Measur.						1.035	0.990	0.866	1.473

a = T b = S	2.1	5.0	6.3	0.29	1.05	5.4	5.3	5.0	7.1
	1.5	5.0	5.8	0.05	1.15	5.3	5.0	5.0	6.5
	4.2	2.6	4.5	0.98	0.41	4.1	5.0	4.2	6.8
	2.1	4.5	4.2	0.41	0.98	4.7	4.9	4.5	6.6
	1.5	2.6	2.7	0.60	0.85	2.7	3.0	2.6	4.1

a = E b = S	5.2	4.5	6.0	0.87	0.57	6.5	6.0	5.2	9.7
	3.1	5.0	5.7	0.58	0.87	6.0	5.1	5.0	8.1
	4.6	4.5	5.1	0.79	0.67	5.9	5.6	4.6	9.1
	2.6	4.5	4.5	0.44	0.96	4.8	4.5	4.5	7.1
	3.1	3.8	4.4	0.80	0.66	4.9	4.3	3.8	6.9

Average of Predict./Measur.						1.027	1.006	0.913	1.482
